# Visceral leishmaniasis in Somalia: A review of epidemiology and access to care

**DOI:** 10.1371/journal.pntd.0005231

**Published:** 2017-03-09

**Authors:** Temmy Sunyoto, Julien Potet, Marleen Boelaert

**Affiliations:** 1 Institute of Tropical Medicine, Antwerp, Belgium; 2 Médecins sans Frontières Campaign for Access to Medicines, Geneva, Switzerland; Ohio State University, UNITED STATES

## Abstract

Somalia, ravaged by conflict since 1991, has areas endemic for visceral leishmaniasis (VL), a deadly parasitic disease affecting the rural poor, internally displaced, and pastoralists. Very little is known about VL burden in Somalia, where the protracted crisis hampers access to health care. We reviewed evidence about VL epidemiology in Somalia and appraised control options within the context of this fragile state’s health system. VL has been reported in Somalia since 1934 and has persisted ever since in foci in the southern parts of the country. The only feasible VL control option is early diagnosis and treatment, currently mostly provided by nonstate actors. The availability of VL care in Somalia is limited and insufficient at best, both in coverage and quality. Precarious security remains a major obstacle to reach VL patients in the endemic areas, and the true VL burden and its impact remain unknown. Locally adjusted, innovative approaches in VL care provision should be explored, without undermining ongoing health system development in Somalia. Ensuring VL care is accessible is a moral imperative, and the limitations of the current VL diagnostic and treatment tools in Somalia and other endemic settings affected by conflict should be overcome.

## Introduction

The global burden of visceral leishmaniasis (VL) is estimated at 0.2 to 0.4 million cases, resulting in 50,000 deaths every year [1]. Eastern Africa is the second-highest-burdened region, after the Indian subcontinent [2]. VL suppresses the immune response, and epidemics in populations affected by malnutrition or displacement can be severe [3,4]. This deadly parasitic disease has been mainly reported in parts of southern Somalia [5,6], though data from Somalia are scarce and the true magnitude of the VL burden remains unknown.

Somalia was conflict-ridden even before the state implosion in 1991, and its health indicators are among the worst in the world [7,8]. With a high burden of infectious disease [9,10] and weak surveillance systems, outbreaks are commonplace [11]. The United Nations–backed government is still struggling to exert control beyond the capital (Mogadishu) and urban towns, while most of the VL-endemic areas in southern Somalia are controlled by al Shabaab, an al Qaeda-affiliated Islamist movement hostile to international agencies [12]. Health care in these areas is mostly provided by multiple nonstate actors that face great difficulty in accessing those in need [13,14].

Prompt diagnosis plus adequate VL treatment is lifesaving, and in anthroponotic foci, it is the cornerstone of VL control. However, providing VL care is difficult to enact in a fragmented health system [15]. With the establishment of a federal government in 2013, the health system-strengthening agenda has been gaining momentum [16,17], and many unmet needs have been identified [18–20]. In this paper, we review the evidence on the current burden of VL and availability of care in Somalia, from which we derive recommendations for VL control. Our aim is to draw attention to the neglected tropical diseases (NTDs) agenda in fragile state such as Somalia, and hopefully, our recommendations prove useful in similar settings affected by protracted conflict.

## Methods

We searched the MEDLINE (via Pubmed) online database for articles with leishmaniasis and Somalia in the title with no date limit and published up to 31 March 2016 without language restriction. Additional search terms used in Medical Subject Headings (MeSH) were “leishmaniasis, visceral”; “kala-azar”; and “Somalia”. We searched reference lists from these articles by hand to identify other relevant publications. In addition, we searched documents and reports from agencies, institutions, and organizations with projects in or related to Somalia and contacted the authors of this grey literature for additional information. An experience from a VL control project managed by Médecins Sans Frontières (MSF), a private international humanitarian medical non-governmental organization (NGO), was described.

Ethics Statement: All sources/key informants give consent for the article. The study is exempted from ethical review by MSF Institutional Review Board (IRB).

## Results

The literature search yielded 12 papers, all of which were retrieved in full-text and included in the analysis (see Table 1).

**Table 1:** Overview of included studies from the published literature search.

Year, Location	Author	Type of Publication	Summary	Ref
1966, Middle Shebelle	Baruffa	Journal article	Describing the problem of kala-azar in Somalia.	[40]
1968, Middle Shebelle	Cahill KM	Journal article	Describing epidemiology and clinical features of kala-azar patients in east Africa, including in Somalia.	[38]
1971, Middle Shebelle	Cahill KM	Journal article	Description of kala-azar patients seen in Somalia and mapping of the origins.	[39]
1995, Baidoa	Woolhead A	Journal article	Case report of VL in a woman from Baidoa and warning of potential outbreaks because of the war.	[43]
1995, Lower Juba and Middle Shebelle	Shiddo SA et al.	Journal article	Prevalence study using leishmanin skin test (LST) (positive in 26%) and serology (11%) in 438 village inhabitants. Hospital data showed male:female ratio was 3.3:1.	[41]
1995, Lower Juba and Middle Shebelle	Shiddo SA et al.	Journal article	A study to provide baseline data for antibody responses using DAT, IFAT and ELISA- all distinguished well sera from VL patients and healthy controls. DAT is recommended.	[42]
1995, Lower Juba and Middle Shebelle	Shiddo SA et al.	Journal article	Study reporting humoral and cell-mediated immunity amongst VL patients compared to healthy inhabitants.	[45]
1996, Lower Juba and Middle Shebelle	Shiddo SA et al.	Journal article	Study to determine the levels of IgG subclasses and IgE from 22 VL patients from Somalia, compared to healthy controls. Possible diagnostic role for western blot was found.	[46]
2001, northeastern Kenya	Boussery G et al.	Letter	Reported outbreak in 2000 amongst Somali refugees in Dadaab camps in Kenya, with 34 probable or confirmed VL patients. Median age was 15 years. Case fatality rate was 29.4%, and there was concern over situation inside Somalia and the nutrition situation.	[49]
2003, Somalia, northeastern Kenya, southwestern Ethiopia	Marlet MVL et al.	Journal article	In 2000 and 2001, 904 patients with VL were diagnosed from areas which were known as previously nonendemic for VL or had only sporadic cases prior to the epidemic.	[21]
2003, Bakool	Marlet MVL et al.	Journal article	Description of new VL focus in Bakool region, Somalia, an area where VL had not been reported before. In one year, 230 serologically positive cases were diagnosed as VL, with a cure rate of 91.6% with SSG. Additionally, a serological survey of 161 healthy displaced persons found 24 (15%) positive by the LST and three (2%) positive by the DAT.	[22]
2007, Bakool	Raguenaud ME et al.	Journal article	Retrospective analysis of MSF VL data from 2004 to 2006. After an average of 140 admissions per year, a 7-fold increase happened in 2006. 82% of total patients treated for VL originated from Huddur and Tijelow districts. Clinical recovery rate was 93.2% and case fatality rate was 3.9%.	[50]

DAT: direct agglutination test; IFAT: indirect fluorescent antibody test; IgG: immunoglobulin G; IgE: immunoglobulin E; SSG: sodium stibogluconate.

## VL epidemiology in Somalia

The parasite species causing VL in the east-African region belongs to the *Leishmania donovani* complex, and the same species was confirmed in Somalia in 2000–2001 [21]. The presence of sand fly vectors in this region is generally associated with cracks in black cotton clay soil, *Acacia* and *Balanites* woodland, and termite mounds [22]. Semi-arid regions, where the sand fly *Phlebotomus orientalis* is the vector [23], contrast with the savannah and forested areas, where *P*. *martini* and *P*. *celiae* have been incriminated [24]. Exposure to bites mainly happens outdoors—male persons are more at risk because of their cultural roles of herding cattle or forest traversing [25,26]. Women and children are usually infected in and around the house, leading to clusters around VL cases and household contacts [27,28].

In the Bakool region of southwestern Somalia, an entomological assessment identified mainly *P*. *martini* and *P*. *vansomerenae* as potential vectors[22]. These sand flies have their optimal breeding and resting in the ventilation shafts of termite mounds, which are ubiquitous in Somalia[24,29]. Being in the vicinity of termite hills (the eroded or pinnacle type) are thought to lead to exposure [30]. The vector microhabitat in these *Macrotermes* termite mounds is also influenced by various factors, such as moisture, humidity, temperature, and rainfall, all of which are highly variable in different parts of Somalia.

The ecological situation in the endemic foci of VL in the south has yet to be described in depth, as these areas differ from the higher-altitude northern zones. Somalia has a generally arid and semi-arid climate with two seasonal rainfalls. Its southern part is a rugged plateau, crossed by two major rivers, the Jubba and Shabelle (from Ethiopia highlands), with fertile inter-riverine areas. Seasonality of VL vectors (abundance after rainy season) are well established elsewhere [31], but in Somalia, no information exists to date.

There have been no studies on local determinants, risk factors for VL, transmission dynamics, or vector control in Somalia, but the transmission cycle is supposedly human to human, similar to that in Uganda, Sudan, South Sudan, and Kenya [28,32,33]. Though several animals have been suggested as reservoir hosts (dogs, wild mammals such as the Nile rat, mice, gerbils, servals), their role in transmission in the region is unclear [34,35]. Climatic change has led to frequent floods and droughts in the eastern Africa region, which is thought to influence the transmission or epidemic cycle of vector-borne diseases, including that of VL [36,37]. In Somalia, changes in land use, such as agriculture and deforestation, may lead to desertification and provide habitats for VL vectors.

**Fig 1:**
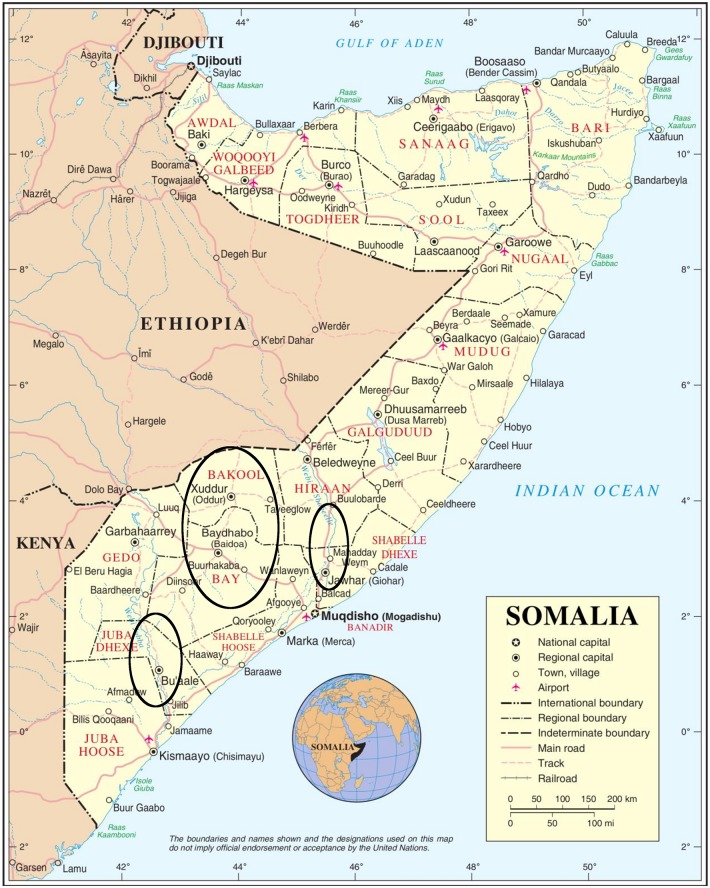
Map of Somalia, with the mark showing approximately the known VL-endemic areas in the country. Adapted from Worldsofmaps.net (under Creative Commons license).

Contrasting with Sudan, where VL was already described in 1904 [38], in Somalia, it was first reported by Penso in 1934 [39], then followed by a case series in 1955 [40]. In the 1960s, Cahill [41,42] and Baruffa [43] mapped the origin of patients. The coastal areas in Lower Juba and Middle Shabelle River were considered endemic [44,45], with the most recent case report coming from Baidoa region [46] (see Fig 1). Few epidemiological surveys were carried out, such as the one in Giohar district, Middle Shabelle showing 26% positivity with the leishmanin skin test (LST)—an intradermal test of the delayed-type hypersensitivity response—and 11% with serology [44]. There are no recent population-based estimates of VL incidence or prevalence in Somalia. An LST survey conducted in 2001 among displaced people in camps around Xuddur, Bakool region revealed a 15% positivity rate, indicating previous exposure to *Leishmania* infection [22]. Different methods, such as immunofluorescence, ELISA, and direct agglutination test (DAT) [47], were used to measure circulating antibodies to provide baseline data and to explore which methods would be the most suitable for diagnosis or for epidemiological population studies in Somalia [48,49].

To date, there have been no reports of VL from the northern parts of Somalia (Somaliland and Puntland zones). There is also no information available in the literature about it, as highlighted in this review. It is clear that the knowledge on VL foci in Somalia is constrained by the country’s emergency situation, which does not allow large epidemiological studies and exhaustive disease mapping to take place. The predictions made by spatial risk maps [50] are consistent, with a significant VL presence in southern Somalia and much less of a presence along the coastal areas of northern Somalia.

VL affects the most marginalised: the rural poor and those lacking access to health services, such as the pastoralists and agropastoralists, who comprise approximately 60% of the population in the south-central zones of Somalia [51]. The socioeconomic impact of VL in a country where almost half the population lives in extreme poverty [52] is not known. In 2000, the confirmation of a VL outbreak among Somalian refugees living in camps in north-eastern Kenya triggered concern about the VL situation inside Somalia [21,53]. From May 2000 to August 2001, 904 VL cases were diagnosed in Kenya, originating from southern Somalia, north-eastern Kenya, and south-eastern Ethiopia. Unusual rainfall patterns, malnutrition, and migration of a population seeking food and security were likely major factors in the outbreak [53]. In this context, an endemic focus was recognized as it was unfolding in parallel in the Bakool region in Somalia, where it was discovered that the “fever and big belly” syndrome that corresponded with the main symptoms of VL had been long known locally [30]. The disease mainly affected children, which had also been observed in Mogadishu hospitals in the early 1990s [44].

## MSF VL control project in Xuddur, Bakool region, 2002–2009

The MSF project in Xuddur started in 2000 as a nutrition program and gradually expanded to a 290-bed health centre by 2008. The VL component commenced when an unusual number of malnourished children did not improve despite proper nutritional support. At first, tuberculosis was suspected as the underlying problem, but VL was later confirmed, as described by Marlet et al. [21,22]. In an 11-month period, 59% of patients presenting a history of fever of at least 1 month, splenomegaly, and wasting tested positive on the DAT and were treated for VL.

A total of 1,671 patients were treated from 2002 to 2006, with a steep increase of cases in late 2005, which later peaked in 2006 with 1,002 new cases and then decreased to 715 and 833 cases in 2007 and 2008, respectively. These numbers do not necessarily reflect the real incidence trend at the population level. The treatment used was injections of sodium stibogluconate (SSG) dosed at 20 mg/kg/day for 30 days. Program data shows an overall case fatality rate (CFR) of 4.5%, while 88% were cured during the period between 2002 and 2008. The defaulter rate improved after the rk39 rapid diagnostic test (RDT) was introduced in 2004, as fewer patients had to wait for DAT tests that had to be performed abroad. Health education on adherence was emphasized, and meals for the caretakers were provided. Better awareness among the population about treatment availability was thought to lead to a shorter duration of sickness before seeking treatment[30].

The programme was negatively affected by the reigning insecurity, which led to repeated evacuations and forced MSF to deploy a remote management approach, in which no presence of international staff could be maintained on the ground anymore. Evaluating the risk after a serious security incident, MSF decided to close the project in Xuddur in 2009 while remaining in other areas of Somalia until the organization pulled out of the country by August 2014 [54].

## Current availability of VL care in Somalia

VL-endemic areas are located in parts of southern Somalia continually mired in conflict. Since al Shabaab rose to prominence in 2008, access to care has been extremely problematic in these areas. At present, there are three facilities able to diagnose and treat VL: two in the Bakool region (Xuddur and Tijeglow) and one in the Bay region (Baidoa). The coverage of these three health centres is not known, as baseline prevalence and population data are missing. The officially reported number of VL cases from the country remains consistent at 400 to 1,000 per year [55,56]. In 2014, an incidence rate of 4.35 per 10,000 inhabitants was estimated by WHO, with an estimated 2–4 underreporting ratio [57].

Adherence to the prolonged VL SSG treatment regimen is a challenge. In the clinic in Baidoa, care is provided on an outpatient basis, and patients who travel from far away have to stay with relatives in town. There is no further referral level for complicated cases beyond the Baidoa clinic. Structured referral is nonexistent. As the VL clinic also provides general health services, its doctors and nurses face a huge workload. It is not uncommon that suspected VL patients travel by their own means to hospitals in Mogadishu to seek care, only to find that no diagnosis tests or drugs for VL are available (M. Dakane, personal communication). In such a context, people tend to use the informal sector, largely composed of privately organized initiatives—pharmacy retailers, traditional healers, and Islamic charities[58,59]. One example is the many children with splenomegaly—one of VL’s main symptoms—who demonstrate burn marks on their stomachs, indicating traditional care-seeking pathways followed before reaching the hospital (G. Elders, personal communication).

Despite the issuance of a 2012 National Guideline for VL in Somalia with support from WHO and various NGOs [60], there is no national control programme in place yet. The precarious security situation remains the stumbling block for active case finding or outreach activity; thus, VL care is in practice restricted to patients who are able to reach treatment centres. Wider community sensitisation on VL prevention and treatment is practically nonexistent. The unstable context also affects procurement and supply of VL diagnostic kits and drugs and the possibility of implementation of vector control measures. Since 2011, WHO has supported the implementing partners with procurement alongside on-the-job training in neighbouring countries (J. A. Ruiz-Postigo, WHO, personal communication).

## Discussion

What is known about VL in Somalia is very limited, as evidenced from our review of the medical literature. Recent global attention to NTDs [61] has not benefited Somalia, where the overall context appears to be a deterrent for action. With the shift from an emergency service delivery approach towards health system building, life-threatening condition like VL are at risk of being further neglected due to emerging, competing priorities in the health sector [62,63].

### Understanding the health system context

The health system in Somalia is a diverse, heterogeneous landscape, mirroring its context [12,64,65]. Apart from al Shabaab’s outright ban on Western agencies [66,67], other factors, such as donors’ counterterrorism legislation and difficulties in negotiating access, have led to cessation or limitation of activity by many NGOs [15,68]. The political economy of aid, subject to politicisation or clan rivalry [69,70], should be well understood in any planning of a health care programme. Thriving private sectors, a weak regulatory environment, urban–rural discrepancies, and the limited reach of state health authorities are features that need to be taken into account.

The Somali social fabric, abiding to customary and Islamic law [71], whereby clans and extended family influence the decision to seek care for illnesses such as VL [72], is important to understand. The cost of health care is almost always borne by households through out-of-pocket expenditure [58]. Although a kinship transfer system—from remittances of Somali diaspora or clan mobilisation—perhaps provides a partial safety net [64], the most vulnerable groups, such as less powerful subclans or nomadic peoples, may not benefit. Additionally, there is urban bias in care provision [16], with the remote, rural areas where VL is endemic being underserved.

VL is unamenable to mass preventive chemotherapy or vaccination; case detection and management is, therefore, crucial. Service delivery through the health system would be the generic mantra in most contexts, but for Somalia, we advocate for exploring nonconservative approaches to mitigate the impact of VL. Local, small-scale, indigenous solutions (rather than nationwide goals) may work better in Somalia, with more focus on local (region or district level) priorities and action.

## Way forward

VL care provision cannot wait until peace returns; with the current climate-related famine threat in the Horn of Africa, alertness and preparedness for another VL outbreak is important for the whole region [50,73]. A mobile team strategy has been implemented successfully in South Sudan (M. den Boer, personal communication)—recruited from local tribes, their tasks include training, health education, bringing drugs and diagnostics, and going to places where there are outbreak rumours to provide immediate assistance. Innovative thinking in improving care would also benefit VL patients. Examples include disease risk mapping using a spatially referenced population database in Somalia [74,75] or use of technical support platforms, such as telemedicine, encompassing teleconsultation and telementoring. The latter has already been deployed in Somalia in paediatric and tuberculosis (TB) services, with encouraging results [76–78]. Surveillance, using up-to-date geographical information on the distribution of VL, can assist in targeting the villages where most patients come from to carry out a more active approach if and when circumstances allow. In a complex, protracted conflict like the one in Somalia, the surveillance system suffers from the breakdown in health services and routine data collection, and without a functional governmental health system, the classical approach to epidemiological surveillance as a centrally operated public sector information management system is not obvious. Therefore, innovative approaches to VL surveillance should be explored, starting with improved coordination of various stakeholders (WHO, NGOs, the community) and proactively building on innovative approaches and new technologies. A few examples that can be considered are using crowdsourced information, event-based or community-based surveillance, and exploiting the digital potential of the Somali community, who are using cell phones and internet on a large scale. WHO has paved the way in this case by proposing the online DHIS2 platform as a uniform and flexible platform that allows various stakeholders to participate in the surveillance endeavour in this complex context[79]. Improving the spread of information and awareness about the disease could be done simultaneously thanks to technological advances in communication tools[80].

Qualified health cadres are lacking at all levels in Somalia [8,81], and local initiatives to bring care closer to the community should therefore be supported. To deal with VL, these capacity-building efforts could be better targeted, with basic in-service skills being provided to the local workers, as opposed to formal qualification. The plan to recruit and train female community health workers [17,81], if rolled out in endemic areas of southern Somalia, could include detection of suspect VL cases. Increasing awareness of VL among the population and availability of care are as important as improving the quality of curative service itself. Working with various actors in the complex backdrop of health care provision is labour-intensive, but it may provide better outcomes than what has been implemented in Somalia in the last decades. Commitment from the local communities, through their own structure, would be crucial in ensuring access to care for this deadly disease.

Looking at the local dynamics through a different lens would help in adapting how care should be organized and delivered. However, there are technical obstacles: the existing tools to diagnose and treat VL in the eastern African region are imperfect and extremely difficult to use in conflict settings with scarcity of health staff. The current treatment option of 30 days’ daily SSG injections or 17 days’ combination of SSG and Paromomycin [56,82] are far from ideal, as not all patients are able to travel to the treatment centres or to afford prolonged in-patient care. A short-course oral treatment for self-administration at home would be a breakthrough in such settings. Likewise, better RDTs with improved accuracy and that differentiate between past and present infections are needed, as current treatment cannot be justified to be given empirically without diagnosis confirmation. Clinical diagnosis by community workers would still require certain training and supervision and should be in conjunction with RDT use. There is a clear gap in the current research and development landscape to invest in user-friendly tools that are easier to roll out in unstable contexts such as Somalia. The operational challenges in conflict-ridden areas like parts of Somalia or South Sudan should also be considered when formulating global research portfolios as well as resource allocations (see Table 2 for complete recommendations).

**Table 2:** Recommendations for addressing VL in Somalia.

For policy makers	**Show awareness and commitment toward VL (and other NTDs) as important causes of ill health and suffering of the Somali people** **Ensure mobilisation of resources to tackle VL through focused and concerted efforts with all stakeholders** **Maintain the policy intent, which includes VL control through macro-, meso-, and micro-level planning in endemic areas** **Commit to ensure security and access for health care workers and programmes**
For programme implementers, NGOs, and support agencies (e.g., WHO)	Continue ensuring availability and access to the VL National Guidelines for health care staff, including through training and supervision Optimizing the reach and coverage of free care provision Ensure availability of needed diagnostic kits and treatment Strengthen the surveillance mechanisms Explore innovative approaches to spread awareness of VL and availability of care Manage VL programme sustainably and toward capacity building Advocate for continuing the provision of access to diagnosis and treatment of endemic clusters of VL and strengthening emergency capacity for outbreak
For research community	Contribute to and lead in building in-country research capacity to enlarge the evidence base of VL in Somalia, including operational and implementation research Identify the most relevant research questions, including those related to disease burden, understanding the economic and social cost of VL, barriers to care, and vector control Identify and innovate in research methodology to address these questions in the context of a difficult-to-access, conflict-affected country Continue to address the gaps in VL epidemiology and VL control knowledge and practices, especially for the east Africa region, including Somalia; accelerate the progress for improved tools to be implemented in the field to overcome limitations of diagnosis and treatment regimens

## Conclusion

To ignore the burden of neglected diseases in conflict-affected areas is not only detrimental to public health, but also to our morals [83]. VL in Somalia should not be left as just another neglected disease in a neglected conflict. Existing tools for VL control—albeit imperfect—should be deployed, their outcomes monitored, and efforts continued to develop better control tools. Innovative strategies—adapted to the stateless context—without undermining the health-system-building process are needed. Addressing VL in Somalia is a moral imperative, as it means averting avoidable deaths for the most vulnerable: the rural poor, internally displaced, and nomadic populations.

**Key learning points**

- Visceral leishmaniasis (VL), fatal without treatment, is known to be endemic in parts of southern Somalia, with outbreaks reported in the past.
- Information on VL in Somalia in the literature is scarce and the only currently feasible control option is provision of diagnosis and treatment.
- Due to the ongoing conflict and difficulty in accessing the people in need, availability of VL care within the country is limited.
- There is a need to stop the neglect of VL in Somalia through innovative strategies and improve emergency preparedness.
- Further research is needed to improve existing diagnosis and treatment tools to be more adapted to be used in such a context.

**Five key papers in the field**

1. Chappuis F, Sundar S, Hailu A, Ghalib H, Rijal S, Peeling RW, et al. Visceral leishmaniasis: what are the needs for diagnosis, treatment and control? NatRevMicrobiol. 2007;5(1740–1534 (Electronic)):873–82.
2. Alvar J, Vélez ID, Bern C, Herrero M, Desjeux P, Cano J, et al. Leishmaniasis Worldwide and Global Estimates of Its Incidence. PLoS ONE. 2012;7(5):e35671.
3. Murray HW, Berman JD, Davies CR, Saravia NG. Advances in leishmaniasis. Lancet. 2005;366(9496):1561–77.
4. Marlet MVL, Sang DK, Ritmeijer K, Muga RO, Onsongo J, Davidson RN. Emergence or re-emergence of visceral leishmaniasis in areas of Somalia, north-eastern Kenya, and south-eastern Ethiopia in 2000–01. Trans R Soc Trop Med Hyg. 2003;97(5):515–8.
5. Raguenaud M-E, Jansson A, Vanlerberghe V, Van der Auwera G, Deborggraeve S, Dujardin J-C, et al. Epidemiology and clinical features of patients with visceral leishmaniasis treated by an MSF clinic in Bakool region, Somalia, 2004–2006. PLoS Negl Trop Dis. 2007;1(1):e85.
